# Group B Streptococcus Bacteremia Mimicking Synovitis, Acne, Pustulosis, Hyperostosis, and Osteitis (SAPHO) Syndrome

**DOI:** 10.7759/cureus.27468

**Published:** 2022-07-29

**Authors:** Fumitoshi Fukuzawa, Takanori Uehara, Shiho Yamashita, Yasushi Hayashi, Masatomi Ikusaka

**Affiliations:** 1 General Medicine, Chiba University Hospital, Chiba, JPN

**Keywords:** referred pain, sternoclavicular joint, group b streptococcus, oligoarthritis, septic arthritis

## Abstract

Group B *Streptococcus* (GBS) causes septic arthritis in healthy adults, and a significant number of GBS septic arthritis cases involve multiple joints. Nevertheless, septic arthritis is commonly monoarticular. Here, we report a case of a 45-year-old man who complained of subacute fever and right shoulder and right buttock pain for three weeks despite undergoing garenoxacin treatment for one week. Although synovitis, acne, pustulosis, hyperostosis, and osteitis (SAPHO) syndrome could be a possible differential diagnosis for this patient, the fever and subacute clinical course could not be explained. Blood cultures revealed the presence of GBS; therefore, he was diagnosed with septic arthritis. After antibiotic treatment for six weeks, his symptoms resolved.

## Introduction

Group B *Streptococcus *(GBS), also known as *S. agalactiae*, mainly colonizes the human intestines; however, it can also colonize the vagina and throat. Although GBS is well known for ascending infections in newborns, it can cause septic arthritis in healthy adults [1]. Recently, GBS is becoming increasingly popular for causing infections in various human tissues, including soft tissues [2]. In general, septic arthritis is monoarticular in healthy adults, but septic arthritis caused by GBS can be oligoarticular or polyarticular, and approximately 50% of GBS septic arthritis cases involve multiple joints [1]. Here, we report a case of a 45-year-old man with GBS oligoarthritis involving the sternoclavicular and sacroiliac joints. Our findings will help physicians make accurate diagnoses in future cases.

This article was previously presented as a poster at the 8th Japan Primary Care Association Meeting of Kantoukoushinetsu on November 17, 2019.

## Case presentation

**Figure 1:**
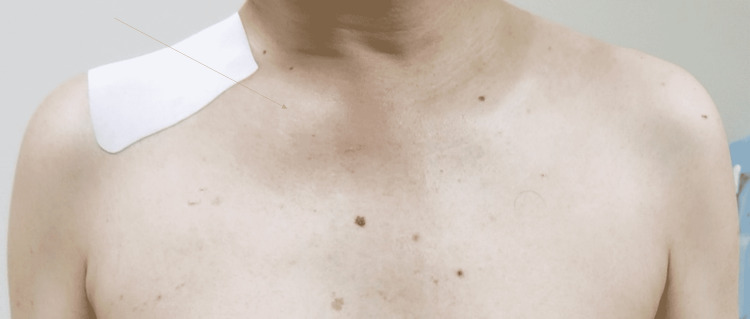
Redness, warmth, and tenderness noted in the sternoclavicular joint

**Figure 2:**
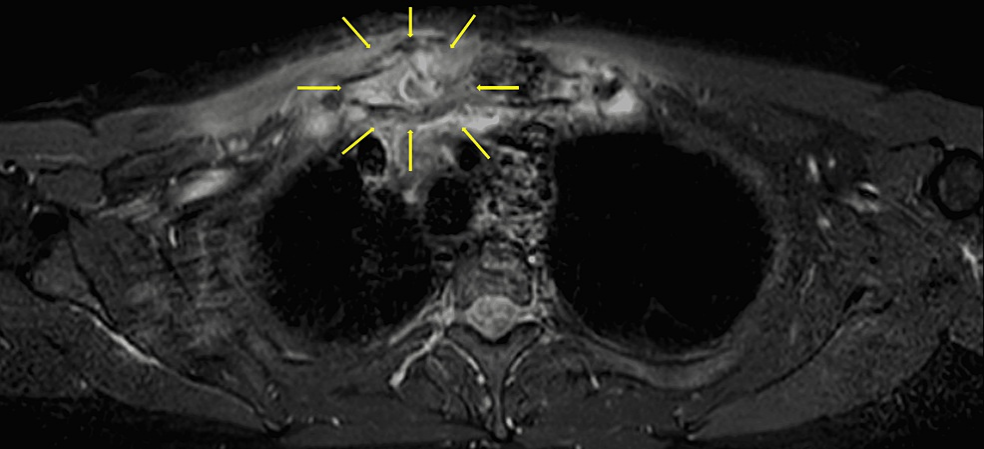
Suspected right sternoclavicular joint arthritis

A 45-year-old man with a three-week history of progressive fever and pain in the right shoulder and right buttock was referred to our hospital. Three weeks before referral, he had received garenoxacin treatment for one week, but there was no improvement in his symptoms. He did not have any relevant past medical, family, or social history. He had engaged in oral intercourse a few days before the onset of these symptoms. Physical examination revealed a body temperature of 38.1℃. The patient experienced shoulder pain on the trapezius ridge and had applied a pain relief poultice to the most painful area (Figure 1). Although no inflammatory signs, such as redness, warmth, and tenderness, were noted on the shoulder joint, he could not move his right shoulder in any direction, and his shoulder pain was exacerbated by active and passive movement of the right shoulder. However, redness, warmth, and tenderness were noted on the sternoclavicular joint (Figure 1). Based on the physical examination findings, referred pain from the sternoclavicular joint was considered [3,4]. Laboratory examination revealed a leucocyte count of 18,300/μL (segmented neutrophils: 80.0%) and a C-reactive protein level of 28 mg/dL. Urine polymerase chain reaction (PCR) assay revealed the absence of *Neisseria gonorrhoeae* and *Chlamydia trachomatis*. Chest to pelvis contrast-enhanced computed tomography and chest magnetic resonance imaging indicated arthritis of the right sternoclavicular joint (Figure 2) and the right sacroiliac joint was done. As the patient had subacute oligoarthritis with sternoclavicular and sacroiliac joint involvement that was non-responsive to antibiotics, synovitis, acne, pustulosis, hyperostosis, and osteitis (SAPHO) syndrome was considered. However, SAPHO syndrome generally has an insidious onset and a chronic course and is not accompanied by high fever [5]. Therefore, the patient’s signs and symptoms could not be explained by SAPHO syndrome. Although multiple joint involvement in septic arthritis is uncommon, we performed blood cultures.

A few days later, the blood cultures revealed the presence of fluoroquinolone-resistant GBS. The patient had no signs of a heart murmur or septic embolism, and transthoracic echocardiography showed no vegetation. Based on the presence of bacteremia and oligoarthritis, the patient was diagnosed with GBS-induced septic arthritis. His symptoms resolved after intravenous antibiotic treatment for six weeks (ceftriaxone empirically, followed by aminobenzylpenicillin per the antibiotic sensitivity results).

## Discussion

SAPHO syndrome generally affects the joints and bones and presents with skin or bowel signs and symptoms typical of the syndrome or consistent with a past history of such manifestations. Our patient did not have any skin lesion or chronic bowel disease according to the diagnostic criteria proposed by Kahn for SAPHO syndrome diagnosis, modified in 2003 [6]. In addition, antibiotic treatment (with fluoroquinolone) was ineffective, and acute oligoarthritis involving the sternoclavicular and sacroiliac joints was noted. In a similar case report about a patient with oligoarthritis, initial management with broad-spectrum antibiotics worsened the patient’s condition; eventually, the patient was diagnosed with reactive arthritis [7]. Therefore, we initially considered that infectious osteitis and bone tumoral conditions were unlikely. Further, as bone and joint manifestations in SAPHO syndrome most often include sternocostoclavicular hyperostosis, anterior chest wall hyperostosis, or sterile osteitis, SAPHO syndrome was considered a possible differential diagnosis. On the other hand, fever is relatively rare (4.5%) in SAPHO syndrome [5], and palmoplantar pustulosis is present in >50% of patients with SAPHO syndrome at the time of musculoskeletal symptom presentation. In SAPHO syndrome cases, the average time from onset to diagnosis is 4.6 years. In general, SAPHO syndrome has an insidious onset and a chronic course and is not accompanied by high fever [5]. The fever and disease course in our patient was not consistent with SAPHO syndrome.

In patients with oligoarthritis involving the sternoclavicular and sacroiliac joints, ankylosing spondylitis may be a possible differential diagnosis. However, the presentation of ankylosing spondylitis is chronic [8], and the disease rarely involves the sternoclavicular joint [9], both of which were inconsistent with our patient’s presentation.

Conversely to SAPHO syndrome, septic arthritis usually manifests as monoarthritis, and oligoarthritis or polyarthritis is rare in previously healthy individuals, except in those with gonococcal arthritis [1]. Therefore, we collected urine specimens for screening *N. gonorrhoeae* and *C. trachomatis* using PCR assay. However, the test results were negative.

Septic arthritis is commonly monoarticular in healthy adults, but GBS-induced septic arthritis involves multiple joints (oligoarthritis or polyarthritis, 47.6% vs. monoarthritis, 52.4%) [10]. GBS can be transmitted through sexual contact [2]. Our patient was previously healthy and sexually active, and invasion of GBS through oral sexual contact was suspected. Some bacteria, such as Gonococcus, Pneumococcus, GBS, and gram-negative bacteria, can cause oligoarthritis [11]. However, oral antibiotics failed to improve our patient’s condition because the isolated organism was resistant to quinolones. Furthermore, oligoarthritis is not a typical presentation in infectious arthritis. Factors such as ineffective antibiotic treatment and oligoarthritis involving the sternoclavicular joint might have led to the diagnostic delay.

## Conclusions

GBS can cause septic oligoarthritis, even in healthy individuals, and can involve multiple joints in some cases. A three-week history of oligoarthritis involving the sternoclavicular and sacroiliac joints, caused by fluoroquinolone-resistant GBS, made us think of the possibility of SAPHO syndrome, which we excluded due to the acute course and the high fever of the patient.

Sternoclavicular joint involvement should be considered when shoulder pain is induced or exacerbated by elevating the upper limbs. Referred pain from the sternoclavicular joint could be to the trapezius ridge. Although no inflammatory signs were seen in the trapezius ridge, referred pain in the trapezius ridge made the patient apply a pain relief poultice to that area.
